# Implementing a Psychotherapy Service for Medically Unexplained Symptoms in a Primary Care Setting

**DOI:** 10.3390/jcm6120109

**Published:** 2017-11-29

**Authors:** Angela Cooper, Allan Abbass, Joel Town

**Affiliations:** 1 Centre for Emotions & Health, Departments of Psychiatry & Family Medicine, Dalhousie University, Halifax, NS B3H 2E2, Canada; 2 Centre for Emotions & Health, Department of Psychiatry, Dalhousie University, Halifax, NS B3H 2E2, Canada; Allan.Abbass@dal.ca (A.A.); Joel.Town@dal.ca (J.T.)

**Keywords:** medically unexplained symptoms, short-term dynamic psychotherapy, family medicine, primary care, emotions, unconscious, somatization, psychotherapy, persistent physical symptoms, ISTDP

## Abstract

Medically unexplained symptoms (MUS) are known to be costly, complex to manage and inadequately addressed in primary care settings. In many cases, there are unresolved psychological and emotional processes underlying these symptoms, leaving traditional medical approaches insufficient. This paper details the implementation of an evidence-based, emotion-focused psychotherapy service for MUS across two family medicine clinics. The theory and evidence-base for using Intensive Short-Term Dynamic Psychotherapy (ISTDP) with MUS is presented along with the key service components of assessment, treatment, education and research. Preliminary outcome indicators showed diverse benefits. Patients reported significantly decreased somatic symptoms in the Patient Health Questionnaire-15 (*d* = 0.4). A statistically significant (23%) decrease in family physicians’ visits was found in the 6 months after attending the MUS service compared to the 6 months prior. Both patients and primary care clinicians reported a high degree of satisfaction with the service. Whilst further research is needed, these findings suggest that a direct psychology service maintained within the family practice clinic may assist patient and clinician function while reducing healthcare utilization. Challenges and further service developments are discussed, including the potential benefits of re-branding the service to become a ‘Primary Care Psychological Consultation and Treatment Service’.

## 1. Introduction

The first aim of this paper is to provide an outline of the development and implementation of an ISTDP service for MUS across two family medicine clinics. The second aim is to describe a service evaluation project and present the preliminary clinical and cost outcome data gathered over the service’s first 18 months. As such, the paper provides a detailed background review that highlights the key issues related to MUS and the use of ISTDP as an evidence-based treatment model. The report details how this service was developed, implemented and adapted over time. There is a detailed focus on the direct and indirect components of the service including the teaching components offered to improve clinicians’ knowledge, skills and competence in working with MUS. The paper then evaluates the strengths of the service, the challenges faced and the potential future developments that may be required both for the MUS service and the wider healthcare system.

Patients with medically unexplained symptoms (MUS) or persistent physical symptoms often present with somatic difficulties for which investigations fail to reveal any pathology (e.g., headache, chest pain, back pain, abdominal pain, dizziness). The blockage of psychological and emotional processes in these patients is thought to result in altered autonomic, endocrine, and immune system activity related to the development of somatic presentations [1]. Specifically, the avoidance of strong emotions has been found to trigger unconscious anxiety, which can be experienced in the form of physical symptoms [2]. Four main patterns of anxiety-driven somatization have been identified: striated muscle tension (e.g., fibromyalgia), smooth muscle activation (e.g., irritable bowel syndrome), cognitive perceptual disruption (e.g., blurred vision), and motor conversion (e.g., paralysis). Further elucidation of these and common examples seen across various healthcare services are presented in Table 1 (adapted with permission from Abbass. Somatization: diagnosing it sooner through emotion-focused interviewing, published by *Journal of Family Practice*, 2005).

The somatization of emotions accounts for a large proportion of family practice visits. Twenty percent of new presentations in primary care and 20–40% of medical outpatient referrals [3,4] present with high rates of somatization into bodily systems including the respiratory, cardiovascular, gastrointestinal, musculoskeletal, neurological and dermatological systems. The health and well-being of patients with these symptoms suffer to a greater extent due to related destructive health behaviors (e.g., smoking, poor diet, patterns of self-neglect, poor medication compliance), leading to disability and ultimately higher overall mortality [5,6,7]. This contributes to enormous healthcare costs, long waitlists and patient/clinician frustration [7,8,9]. In the United Kingdom alone, the annual cost estimate for somatization was £18 billion or CAD 29 billion [10]. Konnopka et al. [11] reviewed health economic studies between 1980 and 2004; they found that costs associated with MUS through hospital admissions, diagnostic procedures and treatment, as well as the indirect costs of lost productivity and absences from work, amounted to USD 18,000 per year per patient (in 2006 values).

This population can be complex, challenging and difficult to engage with supportive medical management [12], and if the psychological and emotional factors underlying these conditions are not addressed, multiple and unnecessary biological investigations, medication trials and specialty referrals may be initiated in an attempt to find an organic cause. One US study, for example, found that 84% of common presentations to an internal medicine clinic yielded no diagnosis, despite expensive investigations [13]. Research has found that MUS patients tend to be over-investigated [14], and the more investigations and referrals they receive, the more difficult it becomes to help them [15]. MUS also accounts for a large portion of excessive and often unnecessary emergency department (ED) use with a significant proportion of patients receiving a ‘not yet diagnosed’ diagnosis. In these cases, unless the underlying emotional factors are dealt with, readmission to the ED is common [16]. 

Given the above, patients and clinicians can become frustrated, or hopeless, due to the lack of a diagnosis and consequently few clear treatment options. This cycle can lead to healthcare misalliances, patient complaints and even lawsuits [17]. Over time, these stressful healthcare interactions begin to impact the wellbeing of clinicians who can start to turn their own emotional responses into somatic difficulties, anxiety, depression and unhealthy coping strategies, such as substance misuse [18]. Taken together, these factors have the potential to lead to burnout, contributing to poorer clinician functioning and heightened risk of medical errors [19]. 

As cases of MUS are rising [14], promising new initiatives aimed at addressing the burden of mismanaged emotional and psychological factors are being developed [14,16]. As a result of such programs, more effective treatment options, improved clinical care, cost-savings, as well as wider healthcare benefits can ensue. These interventions focus on both direct patient engagement and indirect clinician education to build knowledge, skills, confidence and competence in dealing with complex and challenging cases. Through this type of training, clinicians’ emotional self-awareness is enhanced (see ‘Education Curriculum’). 

Intensive Short-Term Dynamic Psychotherapy (ISTDP) is one such treatment option. This therapy model focuses on the bodily experience of emotions and how emotions can convert into bodily symptoms. Specifically, ISTDP seeks to interrupt the buildup of physical symptoms by helping patients tolerate their anxiety, recognize and feel their emotions, develop healthier means of emotional expression and enhance self-care, all of which counteract destructive health-related behaviors. For example, ISTDP has been observed to reduce self-defeating behaviors [6,20,21], and effectively treat anxiety, depression and personality disorders which are commonly co-morbid with physical complaints and excess medical service use [22]. Following a series of randomized controlled trials (RCTs) for medically unexplained pain, ISTDP was found to be more effective than mindfulness-based stress reduction and care as usual [23,24,25,26].

In addition to the findings noted above, ISTDP has been shown to reduce healthcare usage, medication use, hospital and physician visits; studies have found that ISTDP yields significant cost-savings in comparison to ‘treatment as usual’ groups [16,20,27]. A controlled trial of ED patients with MUS found a 69% (*p* < 0.001) reduction in ED visits one year after receiving ISTDP, whereas an untreated control group had a non-significant increase in ED usage [16]. Abbass, Kisely and Rasic et al. [28] assessed the long-term healthcare cost reductions of ISTDP in a tertiary psychiatric service; 890 cases were included with a variety of somatic and psychiatric disorders. This study demonstrated that the healthcare costs associated with these ISTDP patients, reduced to less than those of the general population in the three years after treatment termination, with an average cost reduction of CAD 12,628 per patient. This represents significant cost savings in-line with prior research [27,29]. One of the key mechanisms associated with cost reductions appears to be patients’ level of emotional experiencing; the greater the degree of emotional experiencing, the greater the reduction in healthcare use [20,30,31]. 

### 1.1. Development of an ISTDP Service for MUS in Primary Care

Given that the effectiveness of ISTDP has been established in various clinical settings [16,20,27], a three-year funding bid by the Centre for Emotions and Health (in collaboration with the Department of Family Medicine and Nova Scotia Health Authority) was granted to establish a MUS service with a 1.0 FTE Psychologist, who specializes in ISTDP. MUS patients often fall into a service-provision gap due to their needs being both physical and psychological; locally, these needs are treated by two separate service-provision pathways (i.e., Primary Care for physical needs and Mental Health for psychological needs). Given that MUS patients require an integrated service to address both needs, the current project functions to address this gap.

The service was established in April 2015 across two family medicine clinics to provide consultation, assessment, treatment, teaching and research using ISTDP. The focus of the service is to address unconscious emotional factors and associated patterns of anxiety and defenses that contribute to many of the ‘unexplained’ physical difficulties that often burden family medicine practices (see Table 1). The overarching aims of the project are (1) to pilot the feasibility of embedding ISTDP within the family practice setting and (2) collect and analyze data on the clinical and cost-effectiveness of this approach. This analysis includes evaluating the direct therapeutic outcomes with patients, the impact of teaching and training on clinicians, as well as service and cost-utilization within the wider healthcare system.

### 1.2. Service Context

The MUS service receives referrals from two clinics of the Department of Family Medicine at Dalhousie University in Halifax, Nova Scotia, Canada. Each site provides medical training and placements to family medicine residents and taken together, they serve a population in excess of 4000 patients. Both sites are known locally as Collaborative Care Clinics (CCC), where a number of different professional groups work together in the same clinic to provide a comprehensive package of care for patients. These professionals, forming a team at each of the sites, include, inter alia, doctors, nurses, dietitians and a variety of mental health workers. 

In order to promote the establishment of strong working relationships and the opportunity to take a ‘bottom-up’ approach to knowledge translation regarding MUS, it was crucial to ensure that the MUS Psychologist was fully-integrated into the family medicine clinics, and thus into the existing culture of collaborative interdisciplinary care. Integration was achieved in a number of ways: the MUS Psychologist is located on-site, the psychologist participates in daily interdisciplinary patient meetings, the creation of a clinician-friendly referral letter (see Appendix A), the delivery of monthly teaching workshops, indirect consultations with clinicians, and occasional attendance at departmental team meetings. These integration elements raised the profile of the MUS service within the CCC so that the new assessment and treatment resource could be more effectively utilized.

The daily patient meetings are an important opportunity for MUS knowledge translation and are the foundations of the ‘bottom-up’ approach. This forum is an opportunity to introduce basic psychological concepts to the team, feedback the results of any assessments or treatment progress, and discuss ways to ensure a consistent approach with the patient’s treatment plan. An example of building consistency would include discussions about developing a ‘watch and wait’ approach for those patients referred to the MUS service, meaning, further investigations or changes to current medication are paused whilst the patient is being assessed. This allows the patient and the MUS Psychologist to better determine the role of emotional factors whilst other treatment factors are held constant, thus enabling a clearer picture of the clinical presentation. The daily meetings also give space for team discussions that aid understanding of the dynamics underlying the patients’ symptoms and/or relational difficulties. This process helps to build empathy for the patient and understanding of the life stressors that may have contributed to their difficulties. Time is also spent highlighting clinical cases that may be appropriate for referral, especially when a patient is demonstrating a number of the factors found to be commonly associated with MUS (see Appendix B). Overtime, the daily meetings have become an opportunity to expand the service’s focus from unexplained physical symptoms to incorporate a wider variety of clinical presentations.

### 1.3. Service Documentation

In order to support clinicians to make appropriate referrals and to raise awareness of the service for patients, certain documentation has been compiled. Table 2 highlights the documents developed:

### 1.4. Adapting the MUS Clinic for the Family Practice Setting

The MUS service is new to the family practice clinics, however, due to existing links with Dalhousie University, many of the doctors and medical residents had already received some education about ISTDP and MUS from a local expert in the model who has previously implemented ISTDP services in other departments [16,20,34]. Colleagues from the Centre of Emotions and Health have been providing input to local and international medical education for 15 years and so local medical graduates have had the opportunity to hear about the treatment model and view videotaped material from clinical sessions. This familiarity helped to support the systemic and cultural transition toward utilizing an enhanced biopsychosocial framework.

A variety of educational components are provided and this continues on a monthly basis at both clinics. In the initial phases, the components were designed based on previous MUS research to address known issues and challenges. For example, a previous MUS pilot project [34] emphasized that both patients and clinicians may hesitate to identify emotional contributors due to a tendency to externalize emotional processes [35]. In order to address these challenges, workshops were provided to build knowledge of the links between stress and the body’s physiological response, with the aim of conveying a simple message; stress impacts almost all illnesses whether it is the cause, a contributor or the response. As clinicians have integrated this into their way of thinking, the service has been able to expand the educational components to cover a wider variety of relevant topics. A large part of this curriculum involves building clinicians’ awareness of their own emotions in order to improve their ability to detect emotional processes in patients. Overall, this training aims to develop clinician’s skills to better tolerate complex emotions, manage uncertainty, foster wellbeing, decrease burnout, reduce medical errors and adopt an attitude of positive risk management (see Table 3) [5,7,36]. 

Another important issue to address is the commonly held belief that MUS should only be diagnosed by exclusion, i.e., that these processes can only be diagnosed after all other tests have been ruled out. This does not have to be the case [37]. The central philosophy implemented at the outset of the MUS service is that emotional factors are not assumed to be present, but they are also not assumed to be absent either. Instead, the ISTDP assessment process involves a direct ‘psychodiagnostic’ evaluation in order to ‘rule in or out’ somatic processes by detecting emotional aspects of physical symptoms. In brief, this involves facilitating emotional mobilization and then observing the body’s physiological response to this ‘emotional palpation’ (e.g., presentations such as IBS which are mediated by the sympathetic nervous system). This sort of palpation is analogous to other forms of physical examinations.

This focus on testing and observing bodily processes is fundamental to the ISTDP assessment process. There are a number of potential outcomes based on patients’ response to this ‘emotional palpation’ (see Table 4). The idea that a medical condition could be co-occurring, evolving or subclinical is always held in mind as opposed to an either/or dichotomy between medical and psychological factors. Therefore, attention to psychosocial factors does not preclude vigilance to physical disease and this is a crucial piece that clinicians are required to communicate to their patients. This reduces anxiety about missing a medical problem and enhances the likelihood that patients will accept a biopsychosocial explanation for their symptoms. Indeed, since the service’s inception, a number of medical investigations have been requested by the MUS Psychologist where medically warranted and when emotional factors could not be ‘ruled in’.

### 1.5. Positive Risk Management of MUS

It is recognized that the management of MUS is complex and requires clinical decision making in the context of poorly defined positive and negative risks. Therefore, in order to adopt a ‘rule in’ as opposed to a ‘rule out’ approach, a safe and supportive work environment that encourages positive risk management is crucial. Without this, clinicians are likely to adopt risk aversive practices that are often fuelled by anxiety. In many cases, a medical clinician’s biggest fear is misdiagnosing or missing an illness and whilst some studies have shown a low probability of MUS concealing physical disease, other emerging studies report a 10% incidence of organic disease in those thought to have an MUS presentation [38]. Clinicians need to be able to balance the risks of excessive and unhelpful over-investigation with missed diagnoses. This is why thoughtful positive risk management practices are being promoted and discussed throughout the MUS project cycle. 

Table 3 (adapted with permission from Byng. A whole systems approach to MUS in Plymouth, published by NHS Plymouth, 2009) highlights some of the central ideas promoted by the MUS Psychologist to encourage wider systemic changes [39]. These are designed to promote greater transparency in clinical decision making and to encourage the sharing of risk and responsibility between clinicians and patients. Ultimately, the guidance provided is designed to reduce anxiety and foster decision making in the best interests of patients. This guidance is being implemented across the family medicine clinics, however, this process takes time due to changes in practice that require systemic shifts.

### 1.6. Model of Care

#### 1.6.1. Consultation

Upon request, the MUS Psychologist provides an observation consultation that involves the psychologist watching a clinician–patient visit via live video-link at each site. These only take place if the patient has given consent for the consultation to take place. In this instance, the psychologist may comment upon the interaction between patient and clinician with the aim of improving communication, developing a mind–body understanding, linking psychological phenomena with physiological responses, and using the patient’s bodily responses to guide the process. Often, this type of consultation leads to improved insight and motivation to understand mind–body processes and a referral to the MUS service. In less complex cases, the clinician may feel they have enough knowledge, confidence and skills to work with the patient over a number of visits. In the main, this involves discussing MUS early as a potential differential diagnosis and making unconscious processes more conscious to the patient. This, along with a caring and attuned relationship, can bring symptom relief as patients become more aware of the emotional and/or psychological factors that contribute to their bodily responses.

#### 1.6.2. Assessment 

Once a patient is referred, they receive a two-hour ‘trial therapy’ in which a ‘psychodiagnostic’ evaluation is completed [31,40] The first step explores the nature of the referral and ensures the patient wants to have an interview to evaluate the possible contributions of emotional factors to his or her health problems. With consent gained, the interview may proceed to determine if there is a connection between a patient’s emotions and their symptoms. The initial focus is on what experiences a patient has gone through that have triggered strong emotions and how the patient responds to this focus. From this point, the patient and the psychologist can begin to observe the ways in which emotional factors are physiologically experienced including any physical effects [34]. In addition to the ‘psychodiagnostic’ evaluation, medical, psychiatric and personal histories are taken. A small number of clinical presentations are considered contraindications to ISTDP therapy; these include active substance dependence, active suicidality, organic brain syndromes, severe gastro-intestinal (GI) conditions, psychotic disorders and severe depression [20].

In general, patients are aware of their physical symptoms and this is what they initially seek primary care help for. They are less aware of the emotional drivers beneath those symptoms and, from an ISTDP perspective, avoided and blocked attachment-related emotions (e.g., love, grief, rage, guilt about rage) create the conditions for somatization. Due to early life trauma with attachment figures, current life events activate these old anxiety-provoking feelings especially when those experiences trigger complex feeling states that embody care and love alongside hurt and pain. The attachment processes of deep connection alongside trauma, give rise to feelings of rage and guilt towards loved ones that generally are avoided or buried within the body, because they are either too frightening or unacceptable to be consciously experienced [34].

Habib Davanloo, the originator of ISTDP, emphasized that emotions are biophysiological processes that manifest themselves in universal and specific ways. By studying how emotions are naturally experienced in the body, he could observe what differences occurred in those patients who somatized their emotions. For example, the emotion of rage starts in the lower abdomen, with a sense of heat or energy that rises upward through the chest, neck, arms and finally hands, with associated thoughts and impulses to engage in some form of aggression. Guilt about rage is experienced as a tight chest along with painful feelings, tears and thoughts of regret about the internal experience of rage; as if the rage had actually done harm to another in reality [41]. When such mixed emotions are experienced, they activate the various unconscious pathways of anxiety and somatization described in Table 1. The more the feelings are able to be felt consciously, the less they are being somatized into the body, therefore, if bodily symptoms are present upon the activation of emotions, this allows us to determine the degree to which emotions are contributing to the patient’s presenting physical symptoms.

Previous research has found that there are four main responses that patients can exhibit when emotions are being activated: symptoms can increase or decrease, disappear, or undergo no change at all. These responses are listed in Table 4, along with how the responses are interpreted (adapted with permission from Abbass. Somatization: diagnosing it sooner through emotion-focused interviewing, published by *Journal of Family Practice*, 2005).

In response one, bodily symptoms vary in proportion to the level of emotional activation. For example, stomach pain and gurgling increase as unconscious anxiety rises then settle as anxiety decreases or when the focus moves away from emotional topics; this finding suggests somatization is a contributor to the patient’s IBS. In this case, the patient would be offered ISTDP treatment.

In response two, by focusing directly on the emotions that are mobilized, the patient experiences relief of their symptoms (e.g., patient enters with headache that abates following the experience of their emotions). Here, emotions are likely the cause of the symptoms or at least a contributor. In this case, patients would also be offered ISTDP treatment.

In response three, there is no change in symptoms despite the mobilization of emotions. This suggests that emotional processes are not contributing to symptoms and other testing is appropriate. For example, a patient reports abdominal pain that was thought to be emotional in nature; however, experiencing emotions did not bring relief to this pain. Further medical testing was requested and revealed significant abdominal adhesions that developed from a previous surgical procedure. 

In response four, there is an unclear response. In these cases, a series of follow-up sessions are offered to further evaluate possible emotional contributors. In many cases, other factors such as conscious barriers to engagement, lack of a therapeutic alliance or confusion about the process may need addressing before emotional factors can be directly observed.

For all of these responses, there are important cautionary notes to heed. Both false positives and false negatives are important and can be significantly reduced by the process of repeated emotional mobilization. This gives the therapist more data with which to support or refute their findings, in part, because ongoing changes to chronic symptoms in response to emotional stimulation are unlikely to be due to chance.

#### 1.6.3. Review and Treatment Planning

Following the trial therapy, the findings are reviewed with the patient to determine areas of agreement or disagreement. Future treatment planning depends upon the patient’s response and their ‘psychodiagnosis’; options include further assessment, treatment sessions, no further input and back to the original referrer for further medical testing or other alternatives. For example, if a patient is unmotivated, referral recommendations may include further discussions with their clinician to improve insight and increase motivation to engage in a talking therapy process. Findings are also reviewed with the referrer and documentation is produced to highlight the key aspects of the ‘psychodiagnostic’ assessment process. 

The clinical utility of this process has far reaching implications. In the study’s local context, it is available to be used as a core diagnostic and treatment tool for all medical, surgical and emergency department patients. It is also available as an urgent screening tool for patients with suspected somatic processes who are awaiting ECT, brain implants and general surgery [34].

#### 1.6.4. Treatment

All patients who are appropriate candidates are offered ISTDP therapy. ISTDP is an integrated form of psychotherapy encompassing cognitive, behavioral and emotional elements at varying degrees, tailored to the patient’s capacities and specific needs. The aim of further treatment sessions is to resolve symptoms and work towards optimal health and interpersonal functioning through the continued processing of anxiety-provoking emotions. Typically, these emotions are connected to recent and past attachment traumas and other Adverse Childhood Experiences (ACE). The experience of rage, and crucially, guilt about the rage related to these traumas appears to reduce the need to both avoid emotional closeness and subsequent feeling states (e.g., pain). Self-care is improved and self-destructive tendencies reduce after the guilt is experienced and understood in the context of past experiences. This allows the working through of grief related to losses and termination after a relatively short treatment course [21].

ISTDP has various treatment formats that are tailored to the patient’s presentation and this is determined during the trial therapy and monitored throughout any subsequent sessions. The ‘standard’ ISTDP method aims to address high levels of defensiveness and destructive behavioral patterns that typically stem from attachment traumas. For patients who have low anxiety tolerance, therapy first focuses on developing and building their capacity so that anxiety and emotions are better tolerated. This latter format applies to patients with major depression, dissociation and somatic conditions such as conversion and IBS. This format involves cycles of emotional activation alternating with intellectual reflection and it is called the ‘graded format’ [21,42]. 

Each treatment session is video recorded with the patient’s permission. Key phases of treatment are presented in supervision. This process enables modifications to the therapy process in order to optimize treatment elements (see ‘Ensuring Quality: Use of Deliberate Practice’). An experienced psychiatrist offers supervision across a number of areas including therapist treatment adherence, differential diagnoses, medication effects and factors limiting treatment response.

#### 1.6.5. Education/Teaching 

**Figure 1:**
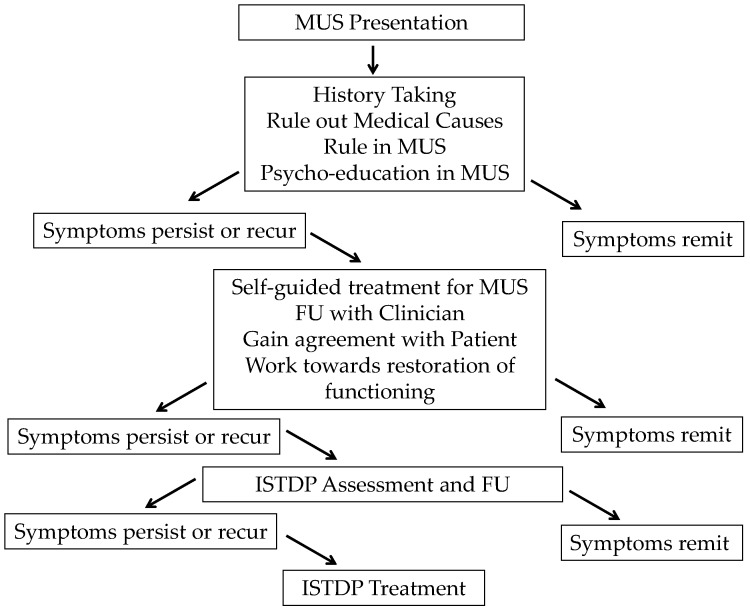
Proposed MUS pathway for development.

The educational component of the MUS service has grown as the service has developed. This growth has been flexible to meet the educational needs of the staff across the two family medicine clinics. Workshops, which originally took place on a bi-monthly basis, moved to monthly at each clinic. In the initial phases, the workshops involved raising awareness of the MUS service, tools to identify MUS presentations, developing an understanding of the treatment process and developing clinicians’ own emotional competencies. Figure 1 highlights the proposed MUS pathway and Table 5 outlines the education plan that is being developed and implemented over the subsequent 18 months of the project. 

In addition to educational workshops, the MUS Psychologist is involved in university teaching programs for medical residents, local and international conferences and various presentations to disseminate the findings from the MUS service.

### 1.7. Research and Evaluation

The MUS service is being developed so that research and service evaluations can be conducted on an ongoing basis. Patients are invited to complete pre-, mid- and post-treatment measures of symptom distress and functioning. The service has access to local healthcare utilization data to measure the extent of its impact and extrapolate cost data. A number of qualitative projects have already been conducted by medical residents which will be published separate to this document. These projects have both educated residents about research methods and enabled them to present at national conferences in order to disseminate preliminary results from the project. It is hoped that further research projects including data on the treatment process will be conducted over the next 18 months with the aim of various publications as well as local, national and international conferences to disseminate our findings further.

### 1.8. Ensuring Quality: Use of Deliberate Practice 

Several elements were used to enable a high-quality treatment service. First, all cases are video recorded for self-review and peer-review. Recordings include visual feedback on both the patient and therapist to allow accurate reflection on both verbal and nonverbal interventions and responses. The videos are viewed by the MUS Psychologist and also by a psychiatrist supervisor in a small group format [43]. Through careful video study, the psychologist may make adjustments for the following session.

Second, weekly and annual videotape based courses are provided to support therapists. In these courses, the method and its underpinnings are reviewed with video of actual treated patients. As part of these, the psychologist is expected to present case material to facilitate the development of teaching skills. 

Third, routine outcome measures are used to provide the patients’ subjective feedback and to allow treatment quality evaluation, as well as standardized self-report measures of anxiety, depression and somatic symptoms. 

## 2. A Service Evaluation

### 2.1. Design

For the purposes of this service evaluation, the outcomes of patients who had been referred and seen in the MUS service were examined, where 6-month follow-up data were available to enable assessment of post-treatment outcomes. All patients included here were consecutively referred to the service from one of the two family medicine clinics. Three primary sources of outcome data were collected to judge the impact of the service: (a) standardized self-report measure of somatic symptom distress administered as part of routine clinical practice; (b) number of primary care clinician visits as a metric of healthcare utilization; and (c) patient and clinician satisfaction questionnaire. 

### 2.2. Measurement of Outcomes 

Patients were asked to complete the Patient Health Questionnaire for somatic symptoms (PHQ-15) [44] prior to the initial assessment appointment and before each subsequent appointment. This is a screening instrument for somatization syndromes; it inquires about 15 somatic symptoms or symptom clusters that account for more than 90% of the physical complaints reported in outpatient settings. 

The number of clinician visits attended by each patient in the 6-month pre-treatment and 6-month post treatment were available from routine data collected from the local family medicine database. This data was collected by a research assistant and the associated clinician billing costs were provided by the family medicine clinic business manager. 

At the end of therapy, both patients and the referring clinician were contacted to give their feedback and experience on the MUS service. The ‘Patient Experience Questionnaire’ covers questions about the patient’s experience of the treating therapist; the service they received; and an open text box to collect qualitative feedback. At the time of data collection, 18 patients had completed this form. The clinician feedback form included a Likert scale from one to ten to rate overall satisfaction with the service (see Appendix G) and an item assessing the current severity of the patient’s MUS. At the time of data collection, seven clinicians had completed this feedback form. Analysis of pre- and post-treatment clinician ratings of severity was not conducted on this sample due to the limited amount of data at this stage.

## 3. Results

### 3.1. Total Sample Description

In the first 18 months of the project, between 1 May 2015 and 31 November 2016, 122 patients were consecutively referred to the MUS service. This equates to 6.7 referrals per month and represents 2.9% of the population served by the two clinics combined. In total, 100 (82%) of those referred were seen for at least one assessment session. They were seen for an average of seven (SD = 6.8) sessions. 

Of the 122 patients referred, 90 (74%) were women, and a mean age of 48 (SD = 16, Range, 18–88). Patients were referred by 55 different clinicians across both clinics; these include family physicians and medical residents. Referrals came from both Mumford (65, 53%) and Spyfield (57, 47%) clinic locations. The average wait between referral and being seen was 32 days. In terms of referral route, 76 (62%) of all referrals were self-directed from clinicians, 27 (22%) came directly from consultation with the MUS Psychologist and 19 (16%) came from information received in the daily patient meetings. Self-directed referrals increased after each educational workshop, suggesting that clinicians feel more confident about their referrals once they have had chance to talk about their case.

Table 6 summarizes the key demographic and baseline characteristics of the 100 patients who were consecutively referred and seen. Of the 100 patients, 73 (73%) were women, 96 completed the PHQ-15, and the majority of patients (66%) scored within the moderate–severe range. In terms of chronicity and persistence of problems, 45 patients (45%) endorsed their physical symptoms to be recurring or continuous and 61 (61%) reported moderate–almost total functional impairment. Those who scored in the moderate range or higher for the GAD-7 (47.4%) and PHQ-9 (30.7%) represent diagnostic cut-offs for anxiety and depressive disorders. The top four referral problems were GI pain or disturbance (*n* = 30, 30%), headache (*n* = 19, 19%), chest pain (*n* = 11, 11%) and chronic back pain (*n* = 10, 10%), comprising approximately two-thirds of all referrals.

### 3.2. Evaluation of Pre–Post Treatment Outcomes (N = 37)

A subsample of 37 treated patients was identified where 6-month post-treatment outcome data was available in order to examine short-term post-treatment outcomes. Of these, 38% attended one session, 46% were deemed treatment completers based on an agreed termination date, and 16% started but did not finish treatment (of these, two patients discontinued due to current adverse life events, two patients did not return with reasons unknown, one patient became too unwell to continue and one patient wanted a different treatment approach). Patients averaged 4.2 sessions (SD 4.2, range 1–23). This includes the initial assessment and any subsequent treatment sessions. 

### 3.3. Quantitative Outcome Results

#### 3.3.1. Pre- and Post-Treatment Questionnaire Measures

A paired samples t-test was conducted to compare the pre- to post-treatment PHQ-15 scores (*N* = 37). The last value carried forward procedure was used for missing data. The results showed a statistically significant decrease (t36 = 3.48, *p* < 0.01, 2-tailed) in physical symptoms with mean scores decreasing from 11.24 (SD = 5.0) to 9.38 (SD = 5.5). Patients’ mean physical symptom score moved from the medium clinical range into the low clinical range, demonstrating a small–medium effect size (Cohen’s *d* = 0.4) [45]. 

#### 3.3.2. Pre- and Post-Treatment Clinician Visits 

Wilcoxon signed-ranks test was conducted using the median as the measure of central tendency. This test indicated that clinician visits 6 months after attending the MUS clinic (median = 3) were significantly lower than the 6 months before (median = 5; ranks Z = −2.13, *p* < 0.05). The data demonstrated a 23% reduction in overall clinician visits representing a cost saving of CAD 44 per patient. A small overall cost saving of CAD 1645 (Based on an average clinician visit cost of CAD 35) was found for the 6-month period post-treatment based on a reduction in clinician visits (155 visits in total) compared to 6-month pre-treatment (202 visits in total). 

### 3.4. Patient Feedback

Following treatment completion, 18 of the 37 patients offered feedback on the service. All patients (100%) indicated that the therapist took their concerns seriously ‘at all times’, eight patients (47%) rated that they had a better understanding of their difficulties ‘at all times’, 11 patients (61%) rated that they felt involved in their treatment choices ‘at all times’, 10 patients (59%) felt they got the help they needed ‘at all times’, and 13 patients (72%) rated having confidence in the therapist’s skills ‘all of the time’. Table 7 highlights some of the feedback received:

### 3.5. Clinician Feedback

Seven clinicians completed feedback forms; the overall satisfaction with the service was rated as 9.1 out of 10 (SD = 0.9, range 8–10), corresponding to the level of ‘very satisfied’. They rated the assessment and therapy process as 8.1 out of 10 (SD 2.2, range 5–10) indicating the level between “satisfied” and “very satisfied”. They rated whether therapy addressed the problems they had referred the patient for as 7.1 (SD 2.9, 2–10), indicating the level “satisfied”. They rated feeling more effective with their patients as 7.1 (SD 2.7, range 2–10), corresponding to the level “satisfied”. Finally, they rated whether they would recommend the service as 9.7 out of 10 (SD, 0.5, range 9–10). 

## 4. Discussion

The aim of this paper is two-fold: to provide an outline of how a brief psychotherapy service for MUS has been implemented across two family medicine clinics and to describe a service evaluation project. From these two areas, we can draw important lessons in how primary care services might begin to effectively integrate the use of psychological assessment and treatment services to further address MUS. 

In the initial 18 months of this pilot project, a total of 122 patients were referred to the psychotherapy service from 55 different clinicians. These figures indicate that this new service was well accessed by a large number of professionals across both family practice sites and confirms a need for psychological services for MUS in the family practice setting. Some of the factors that appear to have contributed to the service being well accessed include: maintaining a visible profile through attendance at daily huddles, being physically located in the clinic, regular teaching workshops and expanding the service to meet the needs of the family medicine context (see ‘Future Service Developments’). These findings support the feasibility of implementing this psychotherapy clinic as a service innovation for MUS in primary care. 

The service evaluation found that patients who accessed the MUS service had a significant reduction in service utilization, as well as self-reported physical symptoms. This preliminary data supports the potential effectiveness of ISTDP for MUS in a family medicine setting, demonstrating small yet significant clinical and cost-related changes. This is useful data given that prior studies have found a lack of research into the treatment of MUS, especially within primary care [10]. In addressing this gap, a UK-based study assessed an innovative primary care service designed to treat complex cases including MUS [46]. They found similar results as this evaluation; decreases in physician visits by 25% and decreases on self-reported measures with effect sizes ranging from 0.4 to 0.6 for patients with MUS specifically. Whilst the UK study included more severe mental health presentations, its aim were broadly similar to the current MUS project and so the outcomes serve as a helpful benchmark when considering the findings presented here. 

Alongside ISTDP, a number of different treatment options are available for MUS. A recent uncontrolled trial [47] analyzed the effectiveness of a brief psychological attribution, emotional awareness and expression therapy (EAET), for patients with chronic pain. They found that changes in both the patients’ attribution of physical symptoms and emotional processes predicted outcome. Specifically, large clinically significant improvements were observed on levels of pain intensity, depression and measures of distress, which lasted for at least 6 months. The authors report that after just five sessions of therapy (one individual and four group sessions), the improvements found substantially surpass those of standard Cognitive Behavioral Therapy (CBT) or Mindfulness acceptance-based therapies. In a larger RCT, EAET was tested against an educational group and CBT, in patients with Fibromyalgia [48]. EAET demonstrated superior results to the education group on a number of measures including, overall symptoms, anxiety, depression and life satisfaction. Similar results were found for EAET and CBT on most but not all of the primary and secondary measures studied. Thus, the authors conclude that, EAET should be considered as an additional but effective treatment option for patients with Fibromyalgia. Whilst CBT is currently the most adequately studied treatment for MUS [49], brief emotion-focused interventions offer the potential for an alternative approach to patients with MUS. Two key components of these treatments include: (1) helping patients move from a physical explanation of their symptoms to a biopsychosocial one, and (2) helping patients become aware of and experience the emotions driving their symptoms which often have their roots in early childhood experiences. 

A great deal of research has now amassed to highlight the strong links between ACE and later life physical and emotional health, with the impact of these events being cumulative (e.g., four or more ACE raise the odds ratio of developing multiple bodily symptoms by 2.7) [50]. The authors of this study assert that the first step in addressing ACE in adulthood is increasing the recognition of their occurrence at the primary care level. This highlights the importance of education within primary care to build understanding of the value in uncovering this type of childhood history in the treatment of MUS [10]. Theories to understand this issue include the long delay between childhood events and the development of health problems in later life, the sensitive nature of the questions that need to be asked and fears around patients’ reactions, most of which are largely unfounded [50]. These issues have brought calls for further research to understand actual clinician–patient encounters to identify and promote the best ways to uncover and talk about these problems [51]. For example, existing research as highlighted some of the common barriers to talking about emotional contributors in MUS that warrant attention [52].

Due to the gaps identified within the MUS literature [50,53], enhancing the training of primary care clinicians became a key focus in the MUS project, to better manage patients who are affected by such adverse experiences. The training efforts employed focused on helping clinicians’ improve their ability to detect and then talk to patient’s about their MUS symptoms whilst making credible and patient-centered biopsychosocial links. These educational sessions appear to have helped clinicians integrate key concepts into their way of thinking and managing MUS. This is demonstrated by the volume of referrals received and the breadth of referrers that utilized the MUS service in the first 18 months. It is likely that, along with providing an integrated treatment service, thinking about emotional causes as a differential diagnosis early in the process of assessment can redirect referrals appropriately and so prevent unnecessary external investigations leading to improved patient care and cost savings. 

The findings from the patient and clinician satisfaction questionnaires provide further evidence of the acceptability and possible effectiveness of this treatment approach. We found that the majority of patients were, satisfied with the service that was provided, developed a deeper understanding of their problems, and felt involved in their own treatment process. Patients reported a positive experience of having the MUS service based within their family practice setting. This view is supported by existing literature that finds patients would choose to involve their family physicians over other practitioners (e.g., specialists) for management of their MUS, due to an established relationship, the continuity of care provided and a perceived lack of judgment [54,55]. Similarly, clinicians’ feedback suggests that they were generally very satisfied with the MUS service. The high scores endorsed on recommending the service to colleagues and patients is a good indicator of how the service is valued and how well it has been accepted by the family physicians and residents.

However, in a local study designed to investigate patients’ thoughts about the MUS service [56], some patients reported that their clinician did not always offer a mind–body explanation for their symptoms. This is in addition to findings that suggest the number of medical tests and treatments prior to a MUS consultation are high [56,57]. Therefore, further targeted education efforts to improve clinicians’ awareness, competence and confidence in MUS will be required going forward (see ‘Future Service Developments’). 

The service evaluation is limited in a number of ways: there was no control group with which to draw comparisons and this limits any conclusions drawn. That said, almost half of the patients referred reported their difficulties to be continuous and more than half endorsed a high level of functional impairment. Previous studies have found that rates of remission in long-standing chronic cases is low [47,53], which lends support to the treatment method used and the reductions found in physical symptoms. 

Additionally, due to the stage of the project, the sample size evaluated was relatively small, and there was an over-representation of women which may have confounded the results in gender-related ways that are unknown at this point. It is not uncommon to have more females than males referred to any psychotherapy service and there are many reasons for this, from differing ways of presenting distress and eliciting care, to a potential tendency for clinicians to identify MUS presentations more in females than males. Though the reasons for this finding are unknown, they are important to consider in the context of any conclusions drawn.

Whilst additional research is needed and further domains of clinical and cost-related changes require evaluation, this is a promising area of development. The feedback gained and service evaluation overall, serves to highlight the potential for integrating the delivery of psychological assessment and treatment services within family medicine clinics to help manage and treat MUS. This approach enables patients to be seen in a relatively short space of time, which could lead to the prevention of more invasive and ineffective treatment interventions that are now known to be associated with poorer outcomes in this population [14]. 

The baseline data evaluated in this service reveals a mixed patient population, many of which endorsed chronic, co-morbid conditions with high rates of medication use, previous therapy attempts, functional impairment and symptom distress. As could be expected from a primary care population, other patients presented with recent onset somatic symptoms, causing milder distress and occasionally lower levels of anxiety and depression symptoms. The heterogeneity of patients seen within primary care could indicate the helpfulness of a care pathway for MUS whereby less intensive yet effective interventions are offered for milder cases, and ISTDP is offered for more refractory conditions or in situations where clarifying the possible contributing role of psychosocial stressors would be beneficial. Future developments of the MUS service will aim to enhance management of and low-level treatment options for primary care clinicians.

### 4.1. Future Service Developments

As a result of the development of greater awareness and comfort in identifying mind–body conditions and emotional health, the MUS service began to receive referrals with more complex co-morbid presentations, including mental health difficulties, personality issues, addiction issues, eating disorders, and chronic physical presentations (including obesity and diabetes). These referrals represent groups of patients who, in part because of their emotional difficulties, have low levels of self-care and often engage in problematic health-related behaviors that contribute to their chronic medical conditions and somatic symptoms. This situation is compounded by a lack of available and accessible psychological treatments. This expansion in complexity is not unsurprising; research has found that, in comparison to patients who have other chronic illnesses, MUS patients report lower quality of life, comparable or greater impairment of physical function, poorer perceived general health and worse mental health [10]. 

Therefore, whilst the MUS service may have initially set out to provide a specific service to a specific group of patients, in reality this idea and even the term itself is potentially problematic. The term MUS does not define a disorder or a diagnosis, in fact the term MUS started more as a description that evolved into a label and then a categorization that is now routinely used to identify a particular patient group [58]. This has led some authors to conclude that MUS describes a predicament, not a specific disorder [59]. MUS as a term is not well liked by patients or clinicians and may promote dichotomous thinking which impacts patient engagement, as well as giving the impression it is a diagnostic entity, which it is not. Therefore, the MUS Psychologist has begun to use the terms mind–body problems and mind–body specialist in reference to the service. These terms seem to be more accepted and better capture the breadth of work that the MUS service provides. 

Flexibility and adaptability have been central to the MUS service’s philosophy and due to a lack of psychological resources for this broad group of clinical presentations, expanding the referral criteria provides a service for those whose needs are not adequately met by either physical or mental health services. Therefore, one important function of the MUS service is providing a bridge between these separate care pathways, enabling a service provision for patient’s benefit which is normally disconnected and inefficient. The MUS service is well-placed to address a greater range of needs as the therapy model used is inherently trans-theoretical with evidence of effectiveness for a broad range of somatic and psychiatric disorders [28], and therefore, this expansion is within the parameters of the treatment frame. Furthermore, within an interdisciplinary team, complex cases with multiple psychiatric comorbidities are typically appropriate referrals for utilizing the skillset provided by psychologists with advanced psychotherapy training. 

Whilst clinicians on the ground can see the importance and relevance of integrating a psychologist with specific skills to provide emotional diagnostics and treatment, further work is required to address the perception that such a specific service may be less relevant to the wider healthcare system. This challenge points us towards further educational opportunities to explain the wide reaching benefits to the healthcare system including the mental health system. When patients receive a direct psychological service in the family practice clinic, the problems can be dealt with at an earlier point which reduces the need for further secondary care or specialist referrals, improves efficiency and reduces healthcare costs.

In terms of indirect interventions, the educational package is constantly adapted to highlight the diverse role of emotions and health. By helping clinicians appreciate the extent to which emotional health impacts the patients they routinely see, we could then begin to build their skills in lower intensity treatment options whilst triaging more complex cases to the MUS service. This, in part, led to the implementation of the service pathway demonstrated in Figure 1, representing a care model that is responsive to service need and demand. By building clinicians’ confidence, competence, skills and knowledge in the management of less complex cases, the MUS service can focus its resources more appropriately whilst staying responsive. In a local service evaluation project [60], clinicians were found to be very willing to engage in further education to help in the management of these diverse presentations. 

The points raised above attempt to highlight the importance of service development to ensure the successful implementation of the MUS service. The key learning points have been the importance of constantly evolving the service in order to flexibly meet the changing needs and demands of a busy family medicine service. It is recognized that, to ensure the continued success of this service, future steps are required, including engagement and education with organizational networks and systems within the local healthcare setting to promote greater sensitivity and awareness to mind–body conditions and emotional health. The service may also consider rebranding itself; this could involve a move away from the MUS label to a more comprehensive title that more accurately captures the service’s activities, such as “Primary Care Psychological Consultation and Treatment Service”.

### 4.2. Clinician Emotional Awareness 

Clinicians surveyed in this subsample are not only open to developing their skills with emotion-linked presentations, but are actively asking for more structured training and an educational framework to facilitate this. Prior research [35] has put forward a strong case for an educational curriculum designed not only to teach healthcare clinicians how to better understand, diagnose and manage the emotional aspects of medical care for patients, but also to develop awareness of their own emotional reactions, personal self-management and emotion-linked difficulties. 

The burden of mismanaged emotions in healthcare is significant, as this paper has outlined. However, this could be offset by helping existing and future clinicians build greater skills in emotional self-awareness. Clinicians who are better able to reflect upon their own emotional reactions are better equipped to diagnose emotional distress and somatization in patients and, as a result, they may prevent excess service use and the additional complications of over investigating and unsatisfactory medical treatments. Additionally, they are likely to better recognize the factors that lead to medical errors and burnout, such as emotional-based reactions to demanding patient behaviors and the helpful or unhelpful management of such reactions thereafter. These factors have all been shown to improve the well-being of medical students [61], enabling clinicians to maintain and even augment the health of both themselves and the healthcare community at large [62]. 

The success of any service that seeks to treat emotion-based disorders, in both a clinical and cost-effective way, is dependent on building this type of knowledge and skills in our primary care and specialty clinicians. These key clinicians, who commonly encounter MUS in their day-to-day clinical work, will have the greatest impact on patient outcomes due to the frequency of contact over time. This leaves both a challenge and an opportunity, since we now know about the existence of emotion physiology and the associated costs of ignoring it both for the patient and all aspects of the healthcare system. Through educational means, the ability to improve the quality of care and reduce service use through early diagnosis of these emotion-linked problems can be realized. However, a cultural shift is needed in order to foster this supportive educational environment along with a culture of self-awareness so that this information is incorporated into and transmitted as a regular part of healthcare education and practice.

### 4.3. Educational Curriculum

In order to address the needs identified above and support the success of the MUS service, further education is required. The educational components include the use of didactic teaching, case-based discussions, emotion-based videotapes, mentoring and supervision. Table 5 highlights the components deemed to be part of an effective curriculum for emotion-linked problems (adapted with permission from Abbass. The case for a specialty-specific core curriculum on emotions and health, published by Royal College Outlook, 2005).

For clinicians who want to specialize in this area, this curriculum may be covered in approximately 32 h: eight hours of didactic seminars, four two-hour small group training sessions and eight videotaped interviews (two hours duration) conducted by the clinician [35]. This would enable a clinician who wants to specialize in this area enough experience to start diagnosing emotional factors in patients, developing new skills to manage challenging patients along with taking care of themselves and potentially teaching others. It is important to note that the goal of this educational curriculum is not about developing clinicians as therapists, rather the aim is to build crucial skills in emotional awareness for self and patients. This can then contribute to positive changes in the understanding and management of the emotional aspects of professional practice; the healthier our clinicians, the healthier, more effective and cost-efficient the whole healthcare system can be. 

### 4.4. Capacity Building 

The model of care presented here, includes regional capacity building for primary care clinicians in communities across our province. The methods of achieving this include a periodic case conference over a tele-health system, periodic video supervision over the tele-health system and attendance at an annual three-day immersion course [63]. The same methods of self-review and routine outcome measures will also be encouraged at local levels enabling system-wide evaluation of treatment effectiveness. How the technique of liaison and local education of these clinicians is delivered will depend on the size of the community and how much time a local clinician can take for such training opportunities.

## Figures and Tables

**Table 1:** Bodily Patterns of Medically Unexplained Symptoms.

Format	Observations during Assessment	Associated Symptoms/Diagnoses
**Striated Muscle**	Progression from hand clenching, arm tension, sighing respirations to whole body tension	Headache, choking sensation, chest pain, fibromyalgia hyperventilation, shortness of breath, panic, back pain
**Smooth Muscle**	Acute or chronic spasm of smooth muscle	Irritable bowel symptoms, abdominal cramps/pain, reflux, nausea, bladder spasm, bronchospasm, coronary artery spasm, hypertension, migraine
**Cognitive-perceptual disruption**	Anxiety affecting the cognitive and, perceptual fields	Visual blurring, blindness, mental confusion, dizziness, pseudoseizures, paresthesias, fainting
**Motor Conversion**	Loss of tone in some or all voluntary muscles	Weakness, unilateral or bilateral paralysis, aphonia

**Table 2:** Documents for Clinicians and Patients.

Document	Description	Audience	Location
**Referral Letter**	This was implemented to facilitate and bring consistency to the referral process. It includes a rating scale of the top three physical symptoms the patient reports.	Healthcare Clinicians	Appendix A
**‘Ruling in MUS’**	This is a clinical tool based on existing research and clinical experience [32]. The higher the score, the greater the likelihood of a MUS. It includes an MUS management algorithm (adapted with permission from Abbass and Schubiner. Hidden from view: a clinician’s guide to psychophysiologic disorders, forthcoming).	Healthcare Clinicians	Appendix B
**‘Common MUS Presentations’**	This checklist can be used to remind clinicians of the presentations most associated with Medically unexplained symptoms (MUS).	Healthcare Clinicians	Appendix C
**‘MUS Information Sheet’**	This visual aid outlines the link between stress, the nervous system and physical symptoms. It categorizes symptoms under the headings of ‘muscular’ ‘nervous system’ ‘neurological’ and ‘other factors’.	Patients	Appendix D
**‘MUS Patient Leaflet’**	This is a patient-friendly information sheet to emphasize the link between life stressors, the body’s response and symptoms. It underscores that the patient’s experience is real but the cause may be stress-based as opposed to medical.	Patients	Appendix E
**‘Might I have a MUS Worksheet’**	This is a worksheet for clinicians to give to patients for homework and to review in follow-up. It invites the patient to link specific stressful events to symptoms and their emotions. It also outlines common personality styles that often accompany MUS presentations (adapted with permission from Schubiner and Betzold. Unlearn your pain, published by mind–body publishing, 2010) [33].	Patients and Clinicians	Appendix F

**Table 3:** Supporting Positive Risk Management Guidance.

1. **Benefits of positive risk management**	Improve function and well-being of patients Protect clinicians and patients from negative risk Provide clinicians with a support structure when making decisions Provide a clear audit trail as justification for difficult decisions Cost reduction and wait time reduction
2. **Potential negative outcomes following excessive referrals**	Whilst it is sometimes necessary to rule out disease, referrals to specialists for investigation can have the following negative effects: Legitimizing the patient’s view of their symptoms as a serious physical illness Subject patients to the risks associated with intrusive investigations Investigations may produce false positives or may pick up on minor abnormalities that will worry the patient (e.g., minor yet normal back abnormalities) Cost for referral and investigations
3. **Ideas for best practice**	Use clinical judgment—if there is not a clear need for further investigations, then arrange to monitor symptoms and reassess after an agreed time, or if symptoms change When making referrals or organizing investigations for those with likely MUS, let patients know that the results are likely to be negative Clearly document negative results and the absence of red flags All appointments should be documented to provide evidence for reasoned inaction or monitoring
4. **Sharing Risk and responsibility**	Discuss cases with local and/or specialist colleagues Gain peer supervision/collaboration in formal clinical meetings or informal discussions to support difficult decisions Sharing risk with patient about why referrals are being or not being made Share relevant information with the patient so that they are able to participate in a shared decision making process Ensure ‘safety-netting’ by developing a contingency plan (e.g., inform colleagues about triggers for a further referral, inform patients about when they should re-present)
5. **Enhance Communication**	Listen to the patient’s concerns and ensure that the patient feels listened to Introduce the patient to potential biopsychosocial causes or exacerbation of symptoms Provide explanations of the symptoms that relate to their understanding and beliefs about the cause of their symptoms—either to support or refute harmful beliefs Communicate clearly with colleagues when referring that a negative result is likely and request a speedy discharge Ensure any relevant biopsychosocial factors which may be important to the assessment process are communicated when referring on

**Table 4:** Response and Interpretation from Emotion-Focused Intensive Short-Term Dynamic Psychotherapy (ISTDP) Assessment.

Response	Interpretation and Response	Cautionary Notes
**1. Symptoms increase by emotion focus or decrease when focus is removed**	Likely diagnosis of somatization. Prescribe ISTDP and monitor symptom response.	False positives may occur due to coincidental symptoms changes in the interview Potential health problems unrelated to somatization may be present
**2. Symptoms improve or removed by emotion focus or emotional experience**	Diagnosis is/was somatization. Monitor gains made in follow-up.	
**3. No change in symptoms**	Somatization unlikely to be the cause, assess for other physical factors.	False negatives due to therapist or patient factors that have not been addressed (e.g., syntonic defenses, medication side effects, incorrect interventions)
**4. Unclear response**	Emotional factors may or may not be present, repeat test. Consider other diagnostic tests or emotion-focused diagnostic testing.	

**Table 5:** Outline of a Curriculum for Emotion-linked Problems.

Teaching Method	Content Areas	Learning Objectives
**1. Didactic Seminars:** **focus on patients**	Emotion physiology and the somatic and behavioral patterns of emotion Review common patterns of somatization through videotape examples of diagnostic procedures	To enable clinicians to see the direct effects of somatization on a patient’s body To differentiate the physical experience of emotions in contrast to somatic processes To develop a deeper understanding of the role of complex emotions in somatic presentations
**2. Didactic Seminars:** **focus on clinicians**	Clinician self-care, time management, professional boundaries, conflict management and general theory on medical error, with emphasis on affective and cognitive dispositions The role of team splitting, black and white thinking, cognitive biases and a clinician’s own style of managing emotions	To build greater self-awareness in order to make unconscious processes conscious To take steps to manage one’s own and patients’ emotional processes more effectively To build towards healthy practices that foster wellbeing
**3. Group Sessions with role play and other experiential exercises**	Videotape case-based discussion to expand on material covered in didactic sessions Group discussions about emotional processes and how to detect them in an assessment interview Focus on specific patient presentations to highlight common yet challenging issues	Exposure to a variety of challenging situations Developing skills in managing such patient challenges in a safe and supportive environment Clinicians can learn what emotional reactions may predispose them to medical error or burnout with certain patients Building a culture of peer consultation and shared problem solving
**4. Videotape of** **Clinician—patient consultations**	Self-reviewing video material Presenting material to supervisor and peers	Enhancing prior learning through direct patient contact and deliberate practice Using this information to gain more direct and self-relevant awareness
**5. Creating a library** **of learning resources**	Videotape library illustrating different somatic patterns during diagnostic interviews and the physiological changes during treatment Literature on research and theory in the area of emotions and health, physician self-awareness and self-care	Enhancing self-directed learning opportunities
**6. Collaborative research opportunities and elective placements**	Specializing in the diagnosis and management of somatizing and personality disordered patients	Developing more enhanced therapeutic and research skills

**Table 6:** Demographic and Baseline Characteristics of Patients seen at least once.

Demographic Variables	*N* = 100	
	M	SD
Age (years)	47.64	11.87
Session No.	6.55	6.83
	***N***	**%**
Female	73	73
Caucasian	91	91
Married	39	39
Single	32	32
Completed University Degree	25	25
Receiving Income Assistance	35	35
Employed Full Time	26	26
Unable to work due to physical or mental health problems	25	25
**Main Presenting Problems**	***N***	**%**
GI Pain or Disturbance	30	30
Headache	19	19
Chest Pain	11	11
Chronic Back Pain	10	10
**Baseline Clinical Characteristics**	**M**	**SD**
PHQ-15 ^a^, sum score (*N* = 96)	12	5.25
GAD-7 ^b^, sum score (*N* = 95)	10	6.63
PHQ-9 ^c^, sum score (*N* = 95)	10	7.62
	***N***	**%**
Psychiatric inpatient admission	17	17
Previous talking therapy (includes psychology or counseling)	66	66
Current psychiatric medication need	59	59
Recurring or Continuous physical problems ^d^	45	45
Moderate—Almost Total functional impairment ^e^	61	61
Not currently exercising	34	34
**Clinical Categories**	***N***	**%**
PHQ-15 Moderate—High physical symptoms (sum score ≥ 10)	66	68.8
GAD-7 Moderate-High anxiety symptoms (sum score ≥ 10)	45	47.4
PHQ-9 Moderately-severe—severe (sum score ≥ 15)	30	30.7

^a^ Patient Health Questionnaire—15 for somatic symptoms; ^b^ Generalized Anxiety Disorder—7 for anxiety; ^c^ Patient Health Questionnaire—9 for depression; ^d^ Based on patient self-rating of chronicity; ^e^ Domains of functional impairment averaged across four areas: social, hobbies, chores and errands as rated by patients.

**Table 7:** Selected Quotes from Patient Satisfaction Questionnaire.

1. “The experience was helpful, although painful/confronting. I think I have enough from our sessions to use to reflect on my anxiety and hopefully continue to address my issues”
2. “The service was very helpful and would recommend to others. Dr. X was excellent with her training that she has, to help people move forward and not to dwell on problems and help dealing with anxiety”
3. “I have been able to change my outlook and relax more concerning my relationships and future job prospects”
4. “I am overwhelmed by the changes that have happened and am grateful, very grateful.”

**Figure A1:**
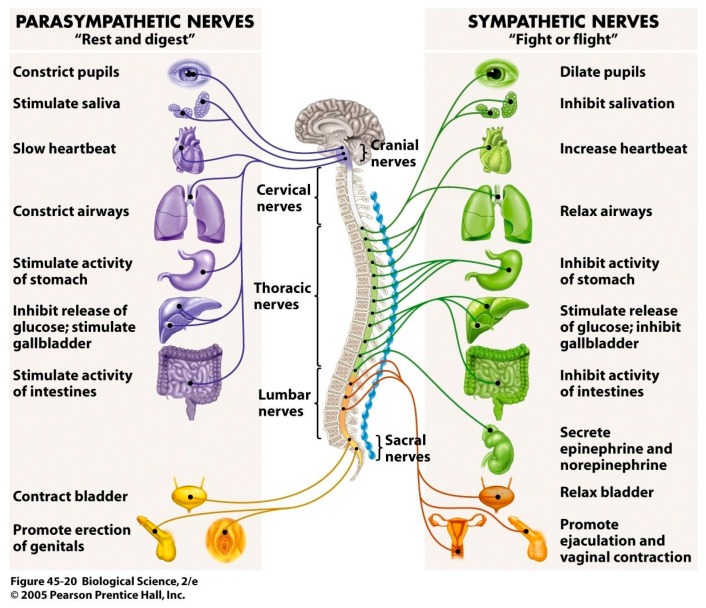
The Autonomic Nervous System.

**Figure A2:**
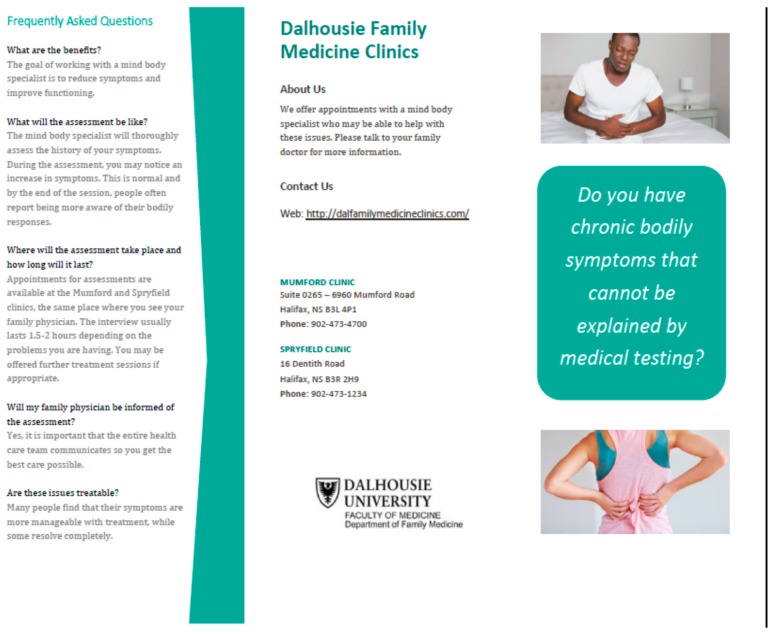
Patient Information Sheet (1).

**Figure A3:**
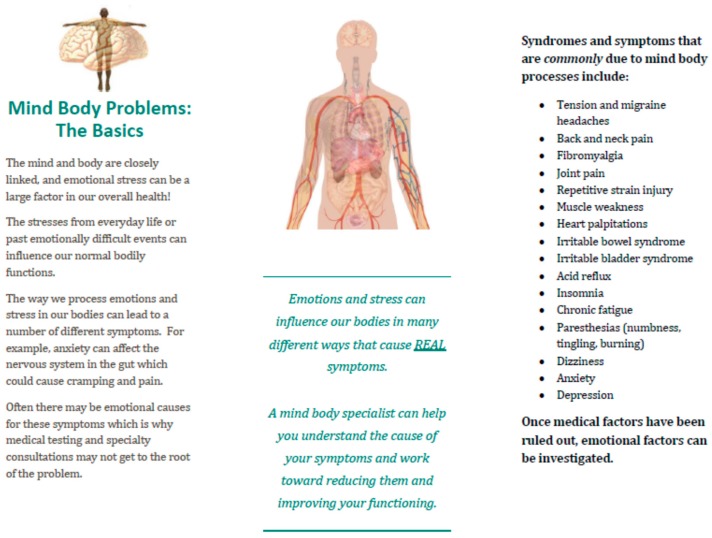
Patient Information Sheet (2).

## Appendix A. MUS Referral Letter

Dear Dr.:

Thank you for seeing this …

1. Please describe current presenting problem and brief history (include onset, duration, and triggers if known)

2. Please identify and rate the primary 1–3 problem(s) that you are referring for: (e.g., anxiety = 7).

Problem		Rating	(1 to 10—Higher score = higher severity)
1.			
2.			
3.			

3. Additional information that may be important to this referral (e.g., risk Hx, previous MH treatment/referrals, medication issues, personality difficulties, substance abuse, Hx of non-attendance, etc. …)

4. Please confirm that you have consulted the referral guidance and you are aware of the service’s referral criteria and contra-indicators. [Referral Guidance]

		(Yes/No)

5. Are you sure this referral is appropriate?

		(Yes/No/Not Sure)

6. Does the patient have an ongoing insurance claim, investigation or pension claim which is connected to their difficulties?

		(Yes/No/Not Sure)

7. Is the patient aware of the referral?

		(Yes/No)

8. Has the patient been give the service’s pamphlet?

		(Yes/No)

9. Please indicate how you made your decision to refer? (Mark “X” for all that apply)

		Clinician Directed
		Consult with shared care clinician
		From discussion in huddle/workshop

Sincerely,

Active Problem(s):

Inactive Problem(s):

Medications:

Surgical History:

Past Medical History:

Family History:

## Appendix B. Ruling in MUS—A Guide

**Table A1:** 10-point Checklist to help rule in MUS *—.

Indicators of MUS	Yes/No	Management Options (See Algorithm Below):
1. Does the patient have what would be considered a common MUS? Do they score 10+ on the PHQ-15?		Low level—FU with clinician in clinic for various: psycho-education, planned writing exercises, physical interventions, mindfulness, other talking interventions etc. More complex—Referral to MUS clinic for ISTDP. Inappropriate for MUS clinic—not motivated, does not agree with mind–body link, not ready yet or other issues need dealing with first (e.g., housing). May require further discussion with clinician. Inconclusive—further medical testing needed.
2. Does their pain/symptoms vary in relation to stress and/or does not conform to known medical distributions (e.g., more pain during work than weekends)?		
3. Have they had a recent emotional stressor?		
4. Is there evidence of anxiety or depressive processes? A score of 10+ on the PHQ-9 and GAD-7 indicate the clinical presence of these disorders.		
5. Has there been an increase in physician visits?		
6. Have medical investigations proven inconclusive?		
7. Have they been unsuccessfully investigated for the same thing in the past?		
8. Is there evidence of Adverse Childhood Experiences?		
9. Do you experience any intense feelings or anxiety when you are with the patient?		
10. When you ask about life stressors does their body respond with anxiety or behaviors that link to the symptoms (e.g., do they get a tight chest in the office and their complaint is chest pain?). Or GI disturbance?		

* Please note, this is a checklist to help guide your clinical opinion, it is based on clinical experience, not official guidance. It does not replace the primary job of ruling out pathology but can help us be more mindful around the need for repeat investigations, medication or specialist referrals. It can also prompt us to have mind–body discussions earlier with our patients in an attempt rule those in. This should decrease some of the resistance to these ideas and improve motivation to engage in talking therapies. The more comfortable we are in having these discussions, the more comfortable our patients will be.

**Table A2:** Examples of Medical Symptoms not Explainable by Emotional Factors Alone.

Symptom	Example of Cause
Severe unilateral headache	Temporal arteritis
Global severe headache with focal neurologic symptoms	Brain tumor
Acute motor weakness	Stroke
Unremitting abdominal pain, nausea or vomiting	Bowel obstruction, Inflammatory bowel disease
Bleeding per rectum	Inflammatory or infectious process
New onset pelvic pain	Inflammatory processes Ectopic pregnancy
Chest pain on exertion	Unstable angina
Persistent fever	Infection or inflammatory condition

## Appendix C. Common Mind–Body Syndromes

**Table A3:** Syndromes Commonly due to Mind–Body Processes.

Chronic Pain Syndromes	Autonomic Nervous System Related Disorders	Other Syndromes
Tension headaches	Irritable bowel syndrome	Insomnia
Migraine headaches	Interstitial cystitis (Irritable bladder syndrome)	Chronic fatigue syndrome
Back pain	Postural orthostatic tachycardia syndrome	Paresthesias (numbness, tingling, burning)
Neck pain	Inappropriate sinus tachycardia	Tinnitus
Whiplash	Reflex sympathetic dystrophy (Chronic regional pain	Dizziness
Fibromyalgia	disorder)	Spasmodic dysphonia
Temporomandibular joint (TMJ) syndrome	Functional dyspepsia	Chronic hives
Chronic abdominal and pelvic pain syndromes		Anxiety
Chronic tendonitis		Depression
Vulvodynia		Obsessive-compulsive disorder
Piriformis syndrome		Post-traumatic stress disorder
Sciatic pain syndrome		
Repetitive strain injury		
Foot pain syndromes		
Myofascial pain syndrome		

NOTE: Almost all of the above disorders can also be caused by structural disease processes so need ruling out in addition to ruling MBS.

## Appendix D. Mind–Body Information Sheet

- Anxiety and stress can affect the body in many different ways, often without you even realizing it.
- Once your doctor has evaluated you and ruled out any serious causes to your problems, we can begin to investigate your levels of stress, tension and the emotional processes which can disturb the normal functioning of your nervous system.
- When your nervous system becomes overactive, it can cause lots of different problems in many parts of your body. Please see the picture below:
- The table below highlights some common symptoms and diagnoses that are often associated with an overactive nervous system.
  **Table A4:** Anxiety Pathways and Associated Symptoms.
  Muscular Issues	Nervous System Issues	Neurological Issues	Other Contributory Factors
  Back Pain	IBS	Migraine	Anxiety
  Chronic pain	Chronic Fatigue	Confusion	Depression
  Fibromyalgia	Stomach and Bowel (e.g., nausea, reflux, diarrhea, constipation)	Weakness	Trauma
  Tension Headache	Bladder Dysfunction	Tinnitus	Current or Ongoing Stress
  Chest Pain	Psoriasis	Dizziness	Adverse Childhood Experiences
  Jaw Pain	Dermatitis	Insomnia	
  Neck Pain	Chemical Sensitivity	Pseudoseizures	
  	Hypertension	Fainting/Falling	
  	Pelvic Pain	Visual Blurring	
  		Drowsiness	
  		Paralysis	
- If you feel you might be suffering with one or more of these symptoms and medical testing has come back negative, you can begin to make a plan with your doctor about the best steps forward to address your difficulties.
- You might want to begin this journey by using our handout and educating yourself about the links between stressful events, your emotional reactions and the symptoms you have developed.
- You might also want to consider a referral to our mind–body specialist who, if appropriate, can offer you an assessment to help you determine if emotional stress is contributing to your symptoms.

## Appendix F. Might I Have Mind–Body Problems?

In order to help you decide if your physical symptoms might be linked to emotionally stressful events, please complete the table below:

**Symptoms**	**Year/Date of Onset**	**Potential Triggering Event (Including Positive or Negative)**	**Emotions That Were Triggered (e.g., Anger/Sadness/Guilt)**
			
			
			
			
			
			
			
			
			

**ANXIETY PATHWAYS**

Next, please circle which anxiety pathways you experience when you think about these emotionally stressful events:

**Muscular Issues**	**Nervous System Issues**	**Neurological Issues**
Muscle tension	Fatigue	Lightheadedness
Jaw clenching	Increased heart rate	Visual blurring
Sighing	Sweating	Blindness
Tension in your head	Dry mouth	Mental confusion
Tension in chest	Cold hands	Dizziness
Hyperventilation	Tingling sensations	Fainting
Tension in shoulders	Bladder spasms	Pseudoseizures
Tension in back	Migraine	Paresthesias
Tension in stomach	Urge to pee	Jelly legs/weakness
Tension in pelvis	Diarrhea	Drowsiness
	Reflux	Hallucinations
	Nausea	Paralysis
		Loss of voice

**PERSONALITY TRAITS**

Next, take a look at the following personality styles which have been found to be related to mind–body symptoms. Please tick any of them that apply to you:

**What Personality Styles Best Describe You:**	**Tick if Apply**
1. Having low self-esteem	
2. Being a perfectionist	
3. Having high expectations of yourself	
4. Wanting to be good and/or be liked	
5. Frequently feeling guilt	
6. Feeling dependent on others	
7. Being conscientious	
8. Being hard on yourself	
9. Being overly responsible	
10. Taking on responsibility for others	
11. Often worrying	
12. Having difficulty making decisions	
13. Following rules strictly	
14. Having difficulty letting go	
15. Feeling cautious, shy, or reserved	
16. Tending to hold thoughts and feelings in	
17. Tending to harbor rage or resentment	
18. Not standing up for yourself	

**WHY DO THESE PERSONALITY TRAITS MATTER?**

- Conflicts between what your mind says and what your body wants can perpetuate mind–body symptoms.
- The traits above are aspects of your conscience—they are things that you might feel obligated to do or ways you might feel obligated to be.
- Most people with mind–body symptom are people who try hard, who care what others think of them, who want to be good and want to be liked. They tend to be conscientious, responsible, and hard on themselves.
- These personality traits are generally found in good people, people you would like to know and be friends with. The problem is that people like this put extra pressure on themselves. They tend to get down on themselves and beat themselves up for their failings.
- When external events and stressors occur and we compound the stress by putting more pressure on ourselves, we are much more likely to develop mind–body symptoms.

**DO YOU NOTICE ANY LINKS?**

- Now that you have completed the tasks above, see if there is a link between the onset of your symptoms, emotionally stressful events, your anxiety channels and your personality style.
- If there are no links, you are unlikely to have a mind–body problem.
- If you find some links, it might be helpful for you to explore the emotional basis of your symptoms in addition to or sometimes instead of further medical investigations in order to begin treating your symptoms.

**WHAT NOW?**

If you would like to investigate these links further, you can discuss these findings with your doctor. Your doctor will help you make a plan to address the underlying cause(s) of your problems.
