# Correction: Cooper, A.; Abbass, A.; Town, J. Implementing a Psychotherapy Service for Medically Unexplained Symptoms in a Primary Care Setting. *Journal of Clinical Medicine* 2017, *6*, 109–141

**DOI:** 10.3390/jcm7030047

**Published:** 2018-03-06

**Authors:** Angela Cooper, Allan Abbass, Joanna Zed, Lisa Bedford, Tara Sampalli, Joel Town

**Affiliations:** 1 Centre for Emotions & Health, Department of Psychiatry & Department of Family Medicine, Dalhousie University, Halifax, NS B3H 2E2, Canada; 2 Centre for Emotions & Health, Department of Psychiatry, Dalhousie University, Halifax, NS B3H 2E2, Canada; Allan.Abbass@dal.ca (A.A.); Joel.Town@dal.ca (J.T.); 3 Dalhousie Department of Family Medicine and Primary Health Care, Nova Scotia Health Authority, Halifax, NS B3J 2R8, Canada; Joanna.Zed@dal.ca; 4 Primary Health Care, Nova Scotia Health Authority, Halifax, NS B3J 2R8, Canada; Lisa.Bedford@nshealth.ca (L.B.); Tara.Sampalli@nshealth.ca (T.S.)

The authors wish to make the following corrections to this paper [1].

The paper has been amended to include three additional authors who made contributions to the service context section of the paper. To correct this, Joanna Zed, Lisa Bedford, and Tara Sampalli have been added as authors. There was an incorrect statistic regarding the number of patients that the two family medicine clinics serve; this has been correctly adjusted from ‘approximately 4000’ to ‘approximately 10,000’. As a consequence of this change, the percentage of patients that accessed the medically unexplained symptoms (MUS) service was changed from 2.9% to 1.2%. A sentence regarding the type of ethical clearance required was added to explain that the study was deemed a quality assurance project by the local ethics committee. Service-related terminology has been changed throughout the document in order to ensure consistency. The acknowledgements section has been updated in order to accurately reflect all contributions made to the paper. These changes have no material impact on the conclusions of our paper.

The corrected author list, affiliation list, and acknowledgements are provided below:

Angela Cooper ^1,^*, Allan Abbass ^2^, Joanna Zed ^3,4^, Lisa Bedford ^4^, Tara Sampalli ^4^ and Joel Town ^2^

^1^ Centre for Emotions & Health, Department of Psychiatry & Department of Family Medicine, Dalhousie University, Halifax, NS B3H 2E2, Canada

^2^ Centre for Emotions & Health, Department of Psychiatry, Dalhousie University, Halifax, NS B3H 2E2, Canada; Allan.Abbass@dal.ca (A.A.); Joel.Town@dal.ca (J.T.)

^3^ Dalhousie Department of Family Medicine and Primary Health Care, Nova Scotia Health Authority, Halifax, NS B3J 2R8, Canada; Joanna.Zed@dal.ca

^4^ Primary Health Care, Nova Scotia Health Authority, Halifax, NS B3J 2R8, Canada; Lisa.Bedford@nshealth.ca (L.B.); Tara.Sampalli@nshealth.ca (T.S.)

* Correspondence: angela.cooper@nshealth.ca; Tel.: +1-902-473-4700

**Acknowledgments:** The corresponding author would like to acknowledge the Department of Family Medicine’s Clinical Investigators who helped with the collation of data, statistical analysis, general administrative support, and scientific advice. Additionally, Pamela Lai, Alex Seal, and Adam Rostis are acknowledged for conducting qualitative research referenced within the paper.

The authors would like to apologize for any inconvenience caused to the readers by these changes.
